# Testing of human papillomavirus in lung cancer and non-tumor lung tissue

**DOI:** 10.1186/1471-2407-12-512

**Published:** 2012-11-12

**Authors:** Antonella Galvan, Sara Noci, Francesca Taverna, Claudia Lombardo, Silvia Franceschi, Ugo Pastorino, Tommaso A Dragani

**Affiliations:** 1 Department of Predictive and Preventive Medicine, Fondazione IRCCS Istituto Nazionale Tumori, Via Amadeo 42, Milan 20133, Italy; 2 Department of Diagnostic Pathology and Laboratories, Fondazione IRCCS Istituto Nazionale Tumori, Milan, Italy; 3 Department of Surgery, Fondazione IRCCS Istituto Nazionale dei Tumori, Milan, Italy; 4 International Agency for Research on Cancer, Lyon, France

**Keywords:** Biomarker, Cancer, HPV, Lung

## Abstract

**Background:**

Risk factors for lung cancer, such as cigarette smoking, environmental pollution, asbestos, and genetic determinants, are well-known, whereas involvement of the human papillomavirus (HPV) is still unclear.

**Methods:**

We examined a series of 100 lung cancer patients from Italy and the UK for the presence of HPV DNA in both lung tumor specimens and adjacent non-tumoral specimens from the same patients. Thirty-five of the most clinically relevant HPV types were assayed using PCR amplification of the highly conserved L1 region of the viral genome followed by hybridization with specific probes.

**Results:**

No HPV was detected in tumor specimens nor in normal lung tissue of any patient.

**Conclusions:**

These data indicate that, in this Western series, HPV is not associated with the risk of lung cancer. Our findings will help refine estimates of lung cancer risk in patients affected by a common viral infection involved in other types of human cancer.

## Background

Human papillomavirus (HPV) is the causal agent of cervical carcinoma and the high-risk HPV types 16 and 18 are consistently detected in about 70% of cervical carcinomas in the world [1]. The involvement of HPV in other types of tumors, such as head and neck squamous cell carcinomas and lung cancer, has been investigated over the years, and while for oropharyngeal cancers an oncogenic role of HPV has been established [2], for lung cancer its role is still uncertain.

A recent meta-analysis highlighted the wide variability in HPV prevalence in primary lung cancer specimens, ranging from 0% to 78.3% worldwide, and noted higher frequencies of HPV in Asian than in European countries [3,4]. A recent study of Italian lung cancer patients reported that none of the assayed cancer tissue samples was consistently positive for HPV, thereby disputing a possible link of HPV with lung carcinogenesis [5]. In the interest of contributing to the debate on a possible role of HPV in the etiology of lung cancer, we examined the presence of this virus in lung cancer tissue and also in the adjacent non-tumoral tissue from 100 European patients.

## Methods

Patients were recruited from three hospitals: Istituto Nazionale Tumori, Istituto Europeo di Oncologia (both in Milan, Italy) and Royal Brompton Hospital (London, UK). Study protocols for recruitment were approved by the ethics committees of the three hospitals. Each subject gave informed consent to the use of their biological samples for research purposes. All patients underwent lung lobectomy and the excised specimens were pathologically examined and stored frozen. In addition, from each patient, a small section of normal lung parenchyma distant from the macroscopic lung cancer tissue was removed at surgery and stored frozen. All the tumors were clinically staged, with a prevalence of stage I patients (54%); 19% of patients were non-smokers (Tables 1–2). Within 5–10 min after removal, the tissue was put in plastic tubes and then frozen at −80 C.

**Table 1:** Characteristics of 100 lung cancer patients assayed for HPV DNA in normal lung tissue and in the cancer tissue

**Characteristic**	**No. of patients**
Country of residence	
*Italy*	87 ^a^
*UK*	13
Age at diagnosis (years)	
*<60*	31
*60-70*	32
*>70*	37
Gender	
*Male*	35
*Female*	65
Histology	
*Adenocarcinoma*	72
*Squamous cell carcinoma*	20
*Other*^*b*^	8
Clinical stage ^c^	
*I*	54
*II*	16
*III*	26
*IV*	3

^a^ 44 patients recruited at Istituto Europeo di Oncologia, Milan, Italy, and 43 at Istituto Nazionale Tumori, Milan, Italy. ^b^ Includes 1 large cell carcinoma, 2 small cell carcinomas, 2 neuroendocrine tumors, 1 poorly differentiated tumor, and 2 not-otherwise-specified tumors. ^c^ Data missing for one sample that was not staged.

**Table 2:** Results of HPV DNA testing on lung cancer and adjacent normal lung tissue specimens and characteristics of smoking habit in 100 lung cancer patients, by tumor histology

**Histology**	**No. HPV-positive / total**		**Never-smokers, no.**	**Ever-smokers**		
	**Cancer tissue**^**a**^	**Normal tissue**		**No.**	**Smoking habit, years, median (range)**	**Cigarettes/day, median (range)**
Adenocarcinoma	0 / 67	0 / 72	15	57	40 (15–71)	20 (5–70)
Squamous cell carcinoma	0 / 20	0 / 20	3	17	45 (20–70)	20 (3–45)
Other	0 / 8	0 / 8	1	7	40 (35–60)	20 (10–40)

^a^ Results from 95 tumoral specimens available for testing.

Genomic DNA was extracted from both tumoral and adjacent non-tumoral lung tissue using the DNeasy Blood & Tissue Kit (Qiagen, Valencia, CA, USA); it was quantified using Picogreen dsDNA Quantitation Kit (Invitrogen, Carlsbad, CA, USA).

For the detection of HPV infection, we used the Clinical Array Technology (CLART) HPV 2 kit (Genomica, Madrid, Spain), which combines highly specific and highly sensitive PCR with the technology of low-density arrays. The method is based on the PCR amplification of a 450-bp fragment within the highly conserved L1 region of the viral genome followed by hybridization with specific probes for each HPV type. This method allows detection of minimal quantities of viral DNA of up to 35 of the most clinically relevant HPV types, including 20 types considered high risk (16, 18, 26, 31, 33, 35, 39, 45, 51, 52, 53, 56, 58, 59, 66, 68, 70, 73, 82 and 85) and 15 types classified as low risk (6, 11, 40, 42, 43, 44, 54, 61, 62, 71, 72, 81, 83, 84 and 89) for cervical cancer.

The whole procedure was performed in two physically separated areas: the pre-PCR area, where samples were prepared and DNA was extracted, and the post-PCR area, where products were amplified and then visualized, and strict procedures were developed to avoid specimen contamination. For each HPV test, a pair of primers permitting the amplification of a fragment of the human CFTR gene was used as a genomic DNA control; this was essential for confirming a negative result, since it indicated the presence of DNA from the patient even if HPV was not found. Also, a pair of primers for the amplification of a modified plasmid was used as a PCR control; this was essential to distinguish between an inhibited amplification reaction and a sample that contained no DNA.

**Figure 1:**
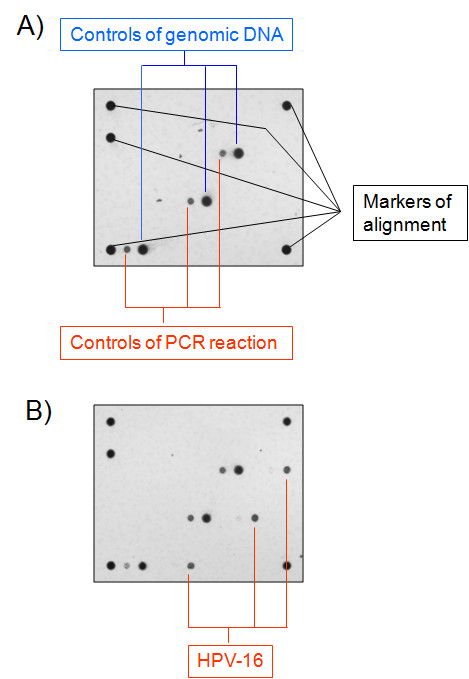
**Array analysis of the presence of HPV DNA in negative (A) and HPV-16 positive (B) controls derived from cervical cytobrush specimens.** For each sample, a pair of primers amplify a fragment of human gene CFTR that is detected in the array as a positive genomic control (Controls of genomic DNA, in blue type), and another pair of primers amplify a modified plasmid that is included in the reaction mix as a control for PCR reaction (Controls of PCR reaction, in red type). Markers are used to align the array for the correct positioning and identification of HPV-specific probes, which are spotted into the array.

We have used previously analyzed cervical cytobrush specimens that were selected as either negative or positive controls. Negative controls derived from patients with negative histological and cytological findings, and resulted HPV negative; positive controls derived from patients with positive histological and cytological findings and found positive for either HPV-6 or HPV-16 genotype (Figure 1).

## Results and discussion

All samples were successfully amplified using the CFTR gene as a positive control for DNA quality and PCR reaction (Figure 1).

Among the lung tumor specimens tested for HPV, five were initially found weakly and doubtfully positive for HPV44 DNA, but repetition of the test with a new assay kit lot indicated that they were negative. Therefore, all the tumor samples were deemed free of HPV infection (Table 2). Among the normal lung tissue specimens, two samples showed borderline positivity for HPV11 in the initial test; however, a repeat analysis using a new lot of the assay kit failed to confirm this finding. Therefore, as for the tumor specimens, also all the normal lung counterparts were considered negative for HPV, independently of their smoking status (Table 2).

Our findings of no HPV DNA in lung cancer specimens in the present Italian-British series is in agreement with Koshiol et al.’s previous study on an independent Italian series [5]. The fact that no HPV DNA was found in adjacent normal lung samples from the same patients confirms results of another recent study in American lung cancer patients [6] but is in variance with other studies carried out in Asian populations, where 4%-to-27% of non-tumoral lung tissue specimens from patients with non-neoplastic lung pathologies was found positive for HPV [7-9].

## Conclusions

Altogether, our findings strongly indicate the absence of a pathogenic role of HPV in lung cancer in a Western population.

## Competing interests

The authors declare no conflict of interest.

## Authors’ contributions

AG wrote the paper and discussed results; SN collected samples, and isolated DNA; FT and CL carried out HPV genotype detection; SF designed the study and analyzed results; UP recruited patients and collected clinical data; TAD supervised all experiments performed as principal investigator. All participants contributed commentary on and corrected the manuscript. All authors read and approved the final manuscript.

## Pre-publication history

The pre-publication history for this paper can be accessed here:

http://www.biomedcentral.com/1471-2407/12/512/prepub
